# Standardized ileal amino acid digestibility and performance indices in pullets and laying hens fed expeller-pressed Canadian prairie soybean meal

**DOI:** 10.1016/j.psj.2024.104081

**Published:** 2024-07-10

**Authors:** Neijat Mohamed, Mingyan Jing, Maryna Plaksii, Shusheng Zhao, Charles M. Nyachoti, Chengbo Yang, James D. House

**Affiliations:** ⁎ Department of Food and Human Nutritional Sciences, University of Manitoba, Winnipeg, MB R3T 2N2, Canada; † Department of Animal Science, University of Manitoba, Winnipeg, MB R3T 2N2, Canada; ‡ Richardson Centre for Food Technology and Research, University of Manitoba, Winnipeg, MB R3T 2N2, Canada

**Keywords:** Glycine max L., ileal digestibility, amino acid, layer pullet and hen, expeller-pressed soybean meal

## Abstract

Soybean meals (**SBM**) from different locations differ in their protein content, subsequently influencing their amino acid (**AA**) profile. In this study, standardized ileal digestibility (**SID**) of AA and growth or production performance were evaluated in pullets and hens fed SBM derived from soybean grown in Western Canada, primarily Manitoba (**MB**) labelled as A-, B- and C-SBM compared with that from Eastern Canada (Ontario, ON-SBM) and contained 38.3 ± 0.44, 38.6 ± 0.61, 39.4 ± 0.49, or 44.0 ± 0.87% CP, respectively. A N-free diet was used to determine basal ileal endogenous losses of AA. The study included the grower, developer, and layer phases (9–12, 13–16, and 44/59–64-wk old birds, respectively). Although a lower (*P* = 0.029) SID for cysteine was noted in the grower phase for the C-SBM compared with other SBM, the developer phase had higher (*P* < 0.05) SID for methionine, phenylalanine, cysteine (more by 4.4, 2.4 and 7.2% units, respectively) on average for SBM samples from MB compared with the ON-SBM. Regardless the source of the SBM, no difference in SID of AA was noted in the layer phase. Overall, in all phases the SID values of most AA in the SBM from MB were comparable with the ON-SBM, which may be linked to higher values of these AA per unit of protein content in the former source. In addition, the growth performance including feed intake, BW gain and feed conversion ratio in pullets, and egg production/quality in layers were similar between treatments. These findings show that the MB-SBM have a comparable feeding value with the ON-SBM, hence represent a suitable alternative protein source for poultry.

## INTRODUCTION

Soybean (*Glycine max* L.) is a primary protein source in typical North American poultry diets. However, substantial variation exists in the composition of soybeans between regions (Ravindran et al., 2014; Mourtzinis et al., 2018), attributable to various factors including genotype, environment and management (Frikha et al., 2012; Rotundo et al., 2016; Ibáñez, 2020). For instance, in Canada, the Prairie soybeans have lower crude protein (**CP**) content compared to those of eastern varieties, on average 37.6 vs*.* 41.1%, respectively (Statistics Canada, 2018; Cober et al., 2023; Shi et al., 2022), similar to trends observed in the north-central and north-western parts of USA (Rotundo et al., 2016; Miller-Garvin and Naeve, 2019). For this reason, the Canadian Prairie soybean has been less valued, with limited use of the resulting soybean meal (**SBM**) as feed ingredient in monogastric diets. Manitoba exhibits a shorter frost-free period (ranging from 65 to 135 d) compared to Ontario ranging from 90 to 190 d (Ort et al., 2022). In this regard, the development of early maturing varieties allowed for the exponential growth in the production of soybean in Manitoba contributing to about 30% of total production in Canada (Statistics Canada, 2018). Despite genetic improvements, Western Canadian soybeans have historically struggled with a low protein content compared to those grown in Eastern Canada, subsequently influencing the development of soybean processing facilities in Manitoba has lagged behind, economically influencing soybean producers and the animal production industry.

Moreover, other studies (Thakur and Hurburgh, 2007; Medic et al., 2014; Mourtzinis et al., 2018) have indicated that, per unit of protein content, soybean with lower CP content have a higher proportion of the five key essential amino acids (**AA**) in poultry diets (i.e., methionine, cysteine, lysine, threonine, and tryptophan). Additional studies in the context of the primary storage forms of protein in soybeans, glycinin (**11S**) and β-conglycinin (**7S**), have quantified higher 11S:7S ratio in the soybeans grown in Western Canada compared with Eastern Canada (Cober et al., 2023); the 11S fraction, being richer in S-containing AA (Kim et al., 2011). These findings indicate that the digestibility values of protein and AA in poultry diets could be influenced by the source of the SBM (de Coca-Sinova et al., 2010; Frikha et al., 2012) demanding a precise knowledge of the AA pattern of the SBM which matches the animal requirement for optimum growth and performance (NRC, 1994; Rostagno and Pupa, 1995; Ravindran and Bryden, 1999). In poultry nutrition, the coefficient of standardized ileal digestibility (SID) of AA in feed ingredients have been widely applied in diet formulation for use as a standard method to estimate the bioavailability of AA (Ravindran and Bryden, 1999; Kadim et al., 2002; Adedokun et al., 2008). However, little to no data are available about the SID of CP and AA for the SBM derived from MB-grown soybean in poultry diets. Further, no study has determined these coefficients for a specific/target age group of birds considering that the AA digestibility change with the age of birds (Li et al. 2003; Adedokun et al. 2008).

The objective of the current study was to determine the coefficient of standardized ileal digestibility of CP and AA of the Western Canadian (Manitoba, MB) soybean meal relative to that from Eastern Canada (Ontario, ON) to explore its potential utilization as an ingredient in diets for pullets and laying hens. The present research also studied the effects of these SBM on growth/production performance of pullets and laying hens.

## MATERIALS AND METHODS

### Animal Care Protocol

This project was conducted at the animal facility at the University of Manitoba, Canada. All experimental procedures were reviewed and approved by the University's Animal Care Committee (Protocol UM# 53960). The birds were cared for according to the guidelines of the Canadian Council on Animal Care (CCAC, 2009).

### Soybean Sources

Soybean seeds with varying levels of CP content were obtained from local suppliers from different regions along the stretch of highway-75 in the southern part of Manitoba (MB) including, St Adolphe, Niverville, Morris and Winkler, and graded to reflect a low, medium, and high CP content. In addition, a composite variety from Ontario (ON), was evaluated which reflected a higher content of CP. The soybean seeds were expeller-pressed to remove the bulk of the oil (rather than solvent extracted) by Delmar Commodities (Winkler, MB, Canada) and Grand Valley Fortifiers (Cambridge, ON, Canada). The resultant SBM was dried and ground. At both plants, the SBM samples were not dehulled. Two metric ton of each grade of SBM were supplied in 2 tote bags, each analyzed individually by obtaining subsamples while the SBM were transferred from a storage-tote bags to a regular-tote bags. The SBM from MB were labelled as SBM-A, SBM-B, and SBM-C in comparison with the ON meal (**ON-SBM**). To reduce the variability in diet composition, the same batch of each SBM type was used in mixing the diets in all studies (for both pullets and layers). The chemical characteristics of the SBM samples were analyzed before the digestibility and performance studies (Table 1).

**Table 1:** Chemical composition of expeller-pressed soybean meals (as-is basis).^1^

Items	SBM-A	SBM-B	SBM-C	ON-SBM
DM (%)	95.2 ± 0.05	95.3 ± 0.83	95.5 ± 0.13	93.1 ± 0.31
CP (%)	38.3 ± 0.44	38.6 ± 0.61	39.4 ± 0.49	44.0 ± 0.87
ME (Poultry, kcal/kg)	3584 ± 36.4	3605 ± 58.8	3623 ± 15.1	3467 ± 42.7
Crude fat (%)	8.69 ± 0.66	9.08 ± 1.12	8.88 ± 0.20	7.28 ± 0.76
Crude fibre (%)	3.99 ± 0.11	3.37 ± 0.04	3.36 ± 0.07	3.39 ± 0.27
Ash (%)	5.33 ± 0.13	5.55 ± 0.22	5.52 ± 0.09	5.49 ± 0.27
Calcium (%)	0.25 ± 0.00	0.25 ± 0.01	0.26 ± 0.00	0.33 ± 0.02
Phosphorus (%)	0.55 ± 0.00	0.61 ± 0.04	0.59 ± 0.01	0.58 ± 0.04
Trypsin inhibitor^2^	10.0 ± 0.73	10.9 ± 0.20	11.2 ± 0.39	14.9 ± 0.22
Lysine/CP^3^	6.74	6.79	6.60	6.23
Indispensable Amino Acids (g/100g)				
Arginine	2.70 ± 0.089	2.73 ± 0.007	2.71 ± 0.009	3.07 ± 0.20
Histidine	1.17 ± 0.011	1.15 ± 0.011	1.15 ± 0.027	1.15 ± 0.034
Threonine	1.56 ± 0.034	1.57 ± 0.004	1.57 ± 0.003	1.62 ± 0.088
Lysine	2.58 ± 0.001	2.62 ± 0.022	2.60 ± 0.004	2.74 ± 0.16
Methionine	0.61 ± 0.021	0.64 ± 0.015	0.62 ± 0.010	0.61 ± 0.027
Valine	1.89 ± 0.029	1.88 ± 0.010	1.89 ± 0.003	2.00 ± 0.12
Isoleucine	1.81 ± 0.036	1.79 ± 0.006	1.82 ± 0.009	1.97 ± 0.12
Leucine	3.02 ± 0.028	3.03 ± 0.013	3.01 ± 0.028	3.29 ± 0.18
Phenylalanine	1.93 ± 0.039	1.94 ± 0.015	1.93 ± 0.030	2.11 ± 0.12
Tryptophan	0.52 ± 0.017	0.54 ± 0.023	0.54 ± 0.014	0.54 ± 0.048
Dispensable Amino acids (g/100g)				
Cysteine	0.58 ± 0.01	0.58 ± 0.01	0.58 ± 0.01	0.56 ± 0.02
Serine	1.98 ± 0.03	1.98 ± 0.00	1.97 ± 0.01	2.13 ± 0.12
Glycine	1.62 ± 0.01	1.61 ± 0.01	1.63 ± 0.01	1.74 ± 0.09
Aspartic acid	4.56 ± 0.06	4.53 ± 0.01	4.52 ± 0.01	4.96 ± 0.32
Glutamic acid	7.07 ± 0.12	6.70 ± 0.04	7.02 ± 0.08	7.98 ± 0.52
Alanine	1.74 ± 0.02	1.74 ± 0.00	1.76 ± 0.01	1.86 ± 0.09
Proline	1.92 ± 0.04	1.92 ± 0.01	1.94 ± 0.02	2.15 ± 0.09
Tyrosine	1.40 ± 0.05	1.33 ± 0.01	1.38 ± 0.05	1.50 ± 0.13
Per unit CP content (%)^4^				
Lysine	6.74	6.79	6.60	6.23
Methionine	1.59	1.66	1.57	1.39
Cystine	1.51	1.50	1.47	1.27
Threonine	4.07	4.07	3.99	3.68
Tryptophan	1.36	1.40	1.37	1.23

1 SBM-A, -B, and -C are soybean meals derived from Manitoba (MB)-grown soybean; ON-SBM = soybean meal derived from Ontario-grown soybean. Sub-samples analyzed in duplicates (mean ± SD).

2 Trypsin Inhibitor unit (TIU) per milligram sample, 1 TIU/mg sample = 1.9 trypsin inhibitor activity (TIA) mg/g (Kakade et al., 1974).

3 Ratio of total lysine (g/100g) to crude protein (g/100g) content (expressed as a percentage).

4 Content of essential amino acids in SBM on relative basis (per unit protein content, as-is basis).

### Experiment 1. Digestibility Study

#### Diets

For the digestibility studies, four experimental diets based on cornstarch-dextrose were formulated to contain 1 of the 4 SBM types (i.e., SBM-A, SBM-B, SBM-C, or ON-SBM) as the sole source of protein for pullets and laying hens. A N-free diet was used to estimate the ileal endogenous losses of CP and AA for pullets (grower and developer birds). Each of the experimental diets contained identical concentration of CP, that is, 18.0, 17.5, and 17.5% as-fed basis, in wk-12 (grower), wk-16 (developer), and wk-44 (layer) phases, respectively (Table 2). To achieve these CP concentrations, the inclusion level of all SBM tested were 46.2, 46.2, 44.9, and 39.8% of diet for the grower phase; 44.9, 44.8, 43.6, and 38.7% of diet for the developer phase; and 45.0, 44.9, 43.7 and 38.7% of diet for the layer phase, for SBM-A, SBM-B, SBM-C, or ON-SBM, respectively. Cellulose (Solka-Floc 10, JRS, North Tonawanda, NY) was used as a source of fiber/filler, particularly in the N-free diet. Titanium dioxide (Sigma-Aldric, Co., St Louis, MO) was included in all diets at 0.30% as indigestible marker for the calculation of AA digestibilities. The ratio of cornstarch, dextrose and oil was constant between diets in each phase. The experimental diets and the N-free diet were fed for 7 consecutive days during the experimental period, that is, wk-12, wk-16, and wk-44, during the grower, developer, and layer phases, respectively. Fresh water and feed were available to all birds for ad libitum intake throughout the experiments. Managed under a semi-controlled housing environment, room temperature ranged between 21 and 24°C in both the pullet and layer phases, and lighting was set to 12L:12D for pullets and 15L:9D for laying hens. The composition of the diets for digestibility assays in the different growth stages of the birds are presented in Table 2.

**Table 2:** Ingredient and nutrient composition of diets for digestibility studies in pullets (wk-12, grower and wk-16, developer) and layers (wk-44) phases (as-is basis).^1^

	Grower (wk-12-old)					Developer (wk-16-old)					Layer (wk-44-old)			
Items (%)	SBM-A	SBM-B	SBM-C	ON-SBM	NFD^2^	SBM-A	SBM-B	SBM-C	ON-SBM	NFD	SBM-A	SBM-B	SBM-C	ON-SBM
SBM	46.2	46.2	44.9	39.8	-	44.9	44.8	43.6	38.7	-	45.0	44.9	43.7	38.7
Corn starch	39.6	39.7	40.8	45.5	80.9	40.4	40.5	41.6	46.1	80.5	30.6	30.7	31.7	35.9
Solka-Floc	5.00	5.00	5.00	5.00	5.00	5.00	5.00	5.00	5.00	5.00	5.00	5.00	5.00	5.00
Dextrose	2.76	2.77	2.85	3.17	5.64	2.82	2.82	2.90	3.21	5.61	3.06	3.06	3.17	3.59
Titanium dioxide	0.30	0.30	0.30	0.30	0.30	0.30	0.30	0.30	0.30	0.30	0.30	0.30	0.30	0.30
Corn oil	1.19	1.19	1.23	1.37	2.43	1.27	1.27	1.31	1.45	2.53	2.26	2.27	2.34	2.65
Biophos^3^	1.31	1.31	1.33	1.42	2.14	1.33	1.33	1.35	1.44	2.14	1.23	1.23	1.26	1.35
Vit-Min premix^4^	1.50	1.50	1.50	1.50	1.50	1.50	1.50	1.50	1.50	1.50	1.50	1.50	1.50	1.50
Limestone	1.61	1.64	1.64	1.55	1.56	1.98	2.01	2.01	1.93	1.93	10.6	10.6	10.6	10.5
Salt	0.46	0.46	0.46	0.47	0.50	0.46	0.46	0.46	0.47	0.50	0.44	0.44	0.44	0.44
***Calculated nutrient***														
ME (Poultry, kcal/kg)	3415	3419	3411	3397	3555	3407	3411	3403	3390	3546	3133	3137	3132	3127
CP	18.0	18.0	18.0	18.0	0.37	17.5	17.5	17.5	17.5	0.37	17.5	17.5	17.5	17.5
Fat	5.90	5.92	5.50	4.89	2.51	5.85	5.86	5.46	4.87	2.61	6.84	6.86	6.50	6.07
Crude fibre	4.13	4.04	3.81	3.90	2.50	4.08	3.99	3.78	3.86	2.50	4.08	4.00	3.78	3.86
Calcium	1.10	1.10	1.10	1.10	1.10	1.23	1.23	1.23	1.23	1.23	4.30	4.30	4.30	4.30
Total phosphorus	0.54	0.58	0.56	0.55	0.45	0.54	0.58	0.55	0.55	0.45	0.52	0.56	0.53	0.53
Av. phosphorus	0.45	0.45	0.45	0.45	0.45	0.45	0.45	0.45	0.45	0.45	0.43	0.43	0.43	0.43
Lysine	1.19	1.21	1.17	1.09	-	1.16	1.17	1.13	1.06	-	1.16	1.18	1.13	1.06
Methionine	0.28	0.30	0.28	0.24	-	0.27	0.29	0.27	0.24	-	0.28	0.29	0.27	0.24
Threonine	0.72	0.72	0.70	0.64	-	0.70	0.70	0.68	0.63	-	0.70	0.71	0.69	0.63
Tryptophan	0.24	0.25	0.24	0.21	-	0.23	0.24	0.24	0.21	-	0.23	0.24	0.24	0.21
Arginine	1.25	1.26	1.22	1.22	-	1.21	1.22	1.18	1.19	-	1.22	1.23	1.18	1.19
Sodium	0.19	0.19	0.19	0.19	0.20	0.19	0.19	0.19	0.19	0.20	0.18	0.18	0.18	0.18
Chloride	0.30	0.30	0.30	0.30	0.30	0.30	0.30	0.30	0.30	0.30	0.29	0.29	0.29	0.28
*Analyzed nutrient*														
DM	92.4	91.2	91.3	90.5	90.6	92.5	92.4	92.5	91.9	90.7	92.9	92.8	92.9	92.4
ME (Poultry, kcal/kg)	3140	3136	3151	3047	2945	3192	3157	3173	3062	2948	2988	2970	2898	2903
CP	17.1	17.2	17.4	18.7	0.64	17.7	17.3	18.2	17.2	0.63	18.0	18.4	17.5	17.6
Fat	5.62	5.73	5.59	4.37	0.93	5.82	5.84	5.39	4.44	1.21	6.61	6.47	5.44	5.15
Crude fibre	5.64	5.15	5.03	4.97	3.10	4.74	4.59	4.67	5.59	3.29	4.70	4.68	4.62	4.25
Calcium	0.93	1.21	1.10	1.22	1.00	1.49	1.38	1.77	1.21	1.55	4.50	4.52	4.40	4.09
Total phosphorus	0.47	0.53	0.54	0.51	0.38	0.50	0.56	0.61	0.50	0.48	0.61	0.50	0.50	0.50

1 SBM-A, -B, and -C are diets containing soybean meals derived from Manitoba (MB)-grown soybean; ON-SBM = diets containing soybean meal derived from Ontario-grown soybean.

2 Nitrogen-free diet.

3 Monocalcium phosphate.

4 Vitamin-mineral premix supplied per kilogram of diet: 11,000 IU of vitamin A; 3,000 IU of vitamin D_3_, 20 IU of vitamin E, 3 mg of vitamin K_3_ (as menadione), 0.02 mg of vitamin B_12_, 0.2 mg of biotin, 6.5 mg of riboflavin, 4 mg of folic acid, 10 mg of calcium pantothenate, 39.9 mg of niacin, 2.2 mg of thiamine, 4.5 mg of pyridoxine, 1000 mg of choline chloride, 125 mg antioxidant (ethoxyquin), 66 mg of manganese oxide, 70 mg of zinc oxide, 80 mg of ferrous sulfate, 10 mg of copper sulfate, 0.3 mg of sodium selenite, 0.4 mg of calcium iodate, 0.67 mg of sodium chloride (salt). Limestone was used as the carrier.

### Birds, Housing and Experimental Procedure

For the pullet phase, a total of 328 Dekalb White pullets (1-day-old) were obtained from a local hatchery in Winnipeg, MB, Canada. Except during the experimental period, the pullets were raised on standard diets based on a corn-SBM formulated to meet nutrient requirement recommendations for Dekalb White (Commercial CS management guide, North American Version L2221-1). For the grower digestibility assay, at the beginning of wk-12 of age, 168 pullets of uniform body weight (mean ± SD, 0.70 ± 0.01 kg/bird) were selected and randomly allocated to 6 replicate cages/treatment and 5 birds/cage and fed 1 of 4 treatment diets, except for the N-free diet group which had 8 replicate cages with 6 birds/cage. The remaining 160 birds were fed the standard grower diet to the end of wk-12, and thereafter fed a standard developer diet, until end of wk-15 of age. Hence, for the developer digestibility assay, at the beginning of wk-16 of age, the 160 pullets of uniform body weight (mean ± SD, 1.03 ± 0.02 kg/bird) were randomly allocated to 6 replicate cages/treatment and 5 birds/cage, except for the N-free diet group which had 8 replicate cages with 5 birds/cage.

In the layer phase, a total of 60 hens (Dekalb White hens, 42-wk-old) of uniform body weight (mean ± SD, 1.74 ± 0.06 kg/bird) were obtained from the poultry unit at the University of Manitoba (Winnipeg, MB, Canada), and allocated to individual cages. The hens were allowed a period of 2 wk to adapt to their individual cages prior to the feeding of the experimental diets, while being maintained on a standard corn-soy-based commercial diets that met or exceeded the breed recommendations guide for Dekalb White (Commercial CS management guide, North American Version L2221-1). Three adjacent cages were treated as an experimental unit, and at the start of wk-44 of age, the 4 experimental diets were randomly assigned to 5 replicate groups of 3 birds/experimental units.

### Sample Collection, Processing, and Analyses

At the end of each experimental period, birds were euthanized by CO_2_ asphyxiation and digesta from entire ileum were flushed with deionized water combined by a small amount of gentle squeezing into clean pre-labeled plastic bags, pooled for the 5 or 6 birds in each replicate pen. Samples were put on ice immediately after collection and stored at −20°C until they were freeze-dried. Freeze-dried ileal digesta, diets and SBM samples (ingredient) were finely ground using coffee grinder.

Dry matter was determined according to standard procedures method 930.15 (AOAC, 2005). Nitrogen was analyzed by the combustion method 968.06 (AOAC, 2005) using a CNS-2000 carbon, N, and sulfur analyzer (LECO Corporation, St. Joseph, MI). The CP values were derived by multiplying the assayed N values by a factor of 6.25. Amino acid contents were analyzed using standardized wet chemistry procedures with an initial step requiring either acid or alkaline hydrolysis. For regular AA (excluding S-containing acids and tryptophan), samples were first hydrolyzed with 6N HCl for 24 h at 110°C under N atmosphere using method 982.30 (AOAC, 2012). Sulfur-containing AA (methionine and cysteine) of samples were analyzed after cold performic acid oxidation overnight before acid hydrolysis (method 985.28; AOAC, 2012). Tryptophan was determined after alkaline hydrolysis with barium hydroxide octahydrate for 20 h at 110°C according to ISO 13904 (ISO, 2005) as described by Nosworthy et al. (2017). Hydrolysates were then processed, after a pre-column derivatization with norvaline as the internal standard, using a Shimadzu Nexera ultra-high performance liquid chromatography (UHPLC) system (Kyoto, Japan) as described by Sá et al. (2023). Amino acid values were calculated from hydrated molecular weight AA. Titanium content was determined after samples were ashed at 600°C for 12 h in a muffle furnace, digested with concentrated nitric acids, diluted and processed using inductively coupled plasma mass spectrometry (ICP–AES Vista, Varian, Palo Alto, CA) according to method 985.01 of AOAC (2005). Trypsin inhibitor (**TI**) level was determined using benzoyl-DL-arginine-p-nitroanilide as a substrate according to method described by Kakade et al. (1974), and TI content was expressed as TI units per milligram of sample (TIU/mg sample). Other chemical components were conducted by Central Testing Labs (Winnipeg, MB, Canada) using standard procedures including crude fat using the hexane extraction method (AOAC, 2005), crude fiber (AOAC, 1990) and minerals (calcium and phosphorus) by inductively coupled plasma mass spectrometry (Varian Inc., Palo Alto, CA) after samples were ashed for 12 h according to (AOAC, 2005) procedures (method 990.08). Gross energy (**GE**) was determined using a Parr adiabatic oxygen bomb calorimeter (Parr Instrument Co., Moline, IL), and metabolizable energy (**ME**) in poultry was derived by calculation as gross energy intake minus gross energy of excreta (pool of feces and urine), considering that gaseous energy loss is negligible (Noblet et al., 2022).

### Experiment 2. Performance Assessment

#### Diets

For the performance assessment study, diets were based on a corn-wheat middling-SBM (SBM-A, SBM-B, SBM-C or ON-SBM) and formulated according to the breed standard for Dekalb White (Commercial CS management guide, North American Version L2221-1) for grower (9–12 wk of age), developer (13–16 wk of age), and layer phases (59–64-wks of age). In each phase, each diet was formulated to contain similar levels of the SBM that is, 28, 26 and 29% of diet for grower, developer, and layer phases, respectively. The nutrient requirement of birds across the dietary treatment was balanced for all nutrients and energy except for AA, especially S-containing AA which were below recommended values (no supplemental synthetic AA were used in the formulation of the diets). The composition of the diets for performance assessment during the different growth stages of the birds are presented in Table 5. Fresh water and feed were available to all birds for ad libitum intake throughout the experiments.

### Birds, Housing and Experimental Procedure

For the pullet phase, a total of 240-day-old Dekalb White chicks were housed in a battery cage system and fed a standard starter diet from 1-d to 8-wk of age; thereafter, the pullets were weighed and randomly assigned to 1 of 4 treatment diets containing the test SBM and grouped into 5 birds/cage with 12 replicate-cages per treatment diet. The study was conducted to the end of wk-16 of age (i.e., wk-9 to -12, the grower stage and wk-13 to -16, developer stage). For the laying hens, a total of 420 layers (Dekalb White hens, 59-wk-old) of uniform body weight (initial average weight ± SD, 1*.*84 ± 0*.*22 kg) were selected from the existing flock at the University of Manitoba poultry facility (Winnipeg, MB, Canada), housed in furnished group cages and fed a commercial/standard layer diet. The 4 experimental diets were randomly assigned to 21 birds/cage with 5 replicate-cages per treatment diet. The study was conducted for a period of 6 wk (59- to 64-wk age of hens). Fresh water and feed were available to all birds for ad libitum intake throughout the experiments.

### Performance Indices

In the pullet phase, feed consumption, BW, and feed conversion ratio (**FCR**) were determined weekly for each replicate cage and average per bird basis (Table 9). Feed consumption was determined weekly as the difference between feed offered and residual feed remaining in feeders and averaged by dividing by the number of hens per replicate-cage. Birds were weighed by cage on a weekly basis. In the layer phase, egg production and weight were recorded daily for each replicate-cage and averaged weekly per on bird-basis (Table 10). Feed consumption in layers was also determined as for pullets, and egg mass and FCR were derived by calculation. In addition, in the last 3 weeks of the experiment, 6 eggs per replicate-cage were collected over the last 3 consecutive days of each week for egg quality assessment (Table 11). For this assessment, intact eggs were first weighed, and the yolk was carefully separated to determine yolk weight using a digital scale. The eggshells were then carefully rinsed with water, with intact eggshell membrane, dried at room temperature for 2 d and weighed. The egg white weight was derived by difference.

### Calculations and Statistical Analysis

Standardized ileal digestibility of CP and AA were calculated using the indicator method using a N-free diet by correcting for basal endogenous losses of CP and AA as described previously (Adedokun et al., 2007; Adeola et al., 2016). The ileal endogenous CP and AA losses were derived for the pullets, grower (12-wk-old) and developer (16-wk-old) phases. For the layer phase, the ileal endogenous CP and AA loss values were obtained by averaging that for the grower and developer phases, herein, determined. In the performance study, the FCR was calculated by dividing weekly feed intake by weekly BW gain (for pullets) or by dividing daily feed intake per bird by daily egg mass (for laying hens). Egg mass was calculated by multiplying percentage hen-day egg production by the average egg weight as described by North and Bell (1990). Digestibility and performance data were analyzed as a one-way analysis of variance using the MIXED procedure of SAS (SAS software 9.4, SAS Institute Inc, 2016 ) in a completely randomized design. The model included the fixed effect of the SBM used in the diet. Weekly data for egg quality indices were analyzed as repeated measures, using the same statistical program. Statistical analysis on the effect of SBM type (treatment), phase, phase x treatment on SID values of 5 key AA were conducted using the Proc Glimmix procedure with a Beta distribution (SAS version 9.4, SAS Institute, Cary, NC). Data were checked for normality prior to analysis. Least-squares means were compared using Tukey's honesty significant difference test. All statements of significance were based on *P* < 0.05.

## RESULTS AND DISCUSSION

### Chemical Composition of SBM Samples and Experimental Diets

The CP content of the SBM samples were (mean ± SD) 38.3 ± 0.44, 38.6 ± 0.61, 39.4 ± 0.49, and 44.0 ± 0.87% for the SBM-A, SBM-B, SBM-C and ON-SBM, respectively (Table 1). Hence, on average, the SBM derived from MB-grown soybean contained 38.8 ± 0.57% CP, which was lower in comparison with the ON meal (44.0 ± 0.87; Table 1). This result agrees with other authors (Barthet and Puvirajah, 2022; Ort et al., 2022) indicating up to 5 percent-units less CP content in the soybean seed/meal from Western Canada compared with those grown in Eastern Canada. These differences in protein content are in part, attributed to the adverse weather conditions such as lower mean temperatures and less precipitation in the former region compared to their counterparts in the latter region (Hertsgaard et al., 2019; Ort et al. in 2022). Other chemical compositions of the bean also change due to climate/environmental conditions, for instance carbohydrates including simple sugars, oligosaccharides, and non-starch polysaccharides content of soybean (Sakla et al., 1988), which in turn can influence the digestibility of nutrients in SBM. Although crude fiber analysis is not a suitable method for characterizing carbohydrates in feedstuffs/diets for poultry, in this study, the MB meal with the lowest CP (38.3%) content had the highest crude fibre content (3.99 ± 0.11%). The result is in agreement with other reports (Grieshop et al., 2003; Serena et al., 2008; García-Rebollar et al., 2016) showing a negative correlation between CP content with crude fiber. In addition, Siegert et al. (2023) showed a negative correlation between AA digestibility and NDF concentration in solvent-extracted SBM from different origins in cecectomized laying hens. The crude fat content in all types of SBM were within the range for expeller-pressed beans, resulting in a meal with higher (8.0–15%) residual oil content (Spragg and Mailer, 2007) than the conventional, solvent extracted SBM of ∼1.5% (NRC, 2012). The SBM samples also had values for Ca and P consistent with those reported in the NRC (1994), which were, on average, 0.27 ± 0.04% and 0.58 ± 0.03%, respectively. The processing conditions for the meal, including temperature and production rates at both plants were unknown. The degree of heat damage in the expeller-pressed samples estimated by the ratio of total lysine to CP ratio, were generally within the acceptable value for protein quality, in the range of 6.2 to 6.6% (Stein et al., 2008); demonstrating that similar processing conditions were used at both processing facilities. However, considering the digestible values for lysine determined in the grower phase (earliest generated values from the time of receipt of SBM samples), the ratio of digestible lysine to CP were 6.19, 6.30, 6.02, and 5.80%, respectively for SBM-A, SBM-B, SBM-C and ON-SBM (data not in Tables). Comparisons made herein, indicate that the analyzable lysine level for ON-SBM with a ratio <6.0, may have been reduced due to heat-damaged (Stein et al., 2008). The TI content in the expeller-pressed meals, analyzed herein, on average 11 TIU/mg sample (20.9 mg/g sample) for the MB SBM and 15 TIU/mg sample (28.5 mg/g sample) for the meal from ON, were higher than those reported for conventional SBM, solvent-extracted in the range of 1.35 to 4.55 mg/g sample (Chen et al., 2020). However, considering an inclusion level of the SBM of up to 27% in poultry diets (Neijat et al., 2014; Ravindran et al., 2014; Erdaw et al., 2017), the final TI content in the diets (not assessed in the current study) would be below the recommended threshold for poultry, 3.9 TIU/mg diet sample (Wedekind et al., 2020). The contents (absolute values) of the five key AA in poultry diets (i.e., methionine, cysteine, lysine, threonine, and tryptophan) of the 3 types of SBM from MB (SBM-A, SBM-B, and SBM-C), were comparable to the ON meal (ON-SBM), despite the differences in CP content (Table 1). In this respect, the individual or the sum of the five key AA, expressed on relative terms (percent unit of CP content), were higher for MB meals compared to the ON meal, consistent with previous reports (Mourtzinis et al., 2018; Aguirre et al., 2022).

For all phases (grower, developer and layer phases), the nutrient contents of the experimental diets for the digestibility study are provided in Tables 2 and 3, and for performance study in Table 4. The amino acid contents of diets are presented in Table 5.

**Table 3:** Analyzed amino acid contents of diets used in digestibility studies in pullets (wk-12, grower and wk-16, developer) and wk-44 layers (as-is basis).^1^

	Grower (wk-12-old)					Developer (wk-16-old)					Layer (wk-44-old)			
Amino acids	SBM-A	SBM-B	SBM-C	ON-SBM	NFD^2^	SBM-A	SBM-B	SBM-C	ON-SBM	NFD^2^	SBM-A	SBM-B	SBM-C	ON-SBM
Indispensable (g/100g)														
Arginine	1.19	1.18	1.18	1.32	0.00	1.17	1.11	1.11	1.15	0.00	1.14	1.17	1.18	1.27
Histidine	0.53	0.49	0.50	0.49	0.00	0.47	0.48	0.53	0.57	0.00	0.75	0.74	0.71	0.67
Threonine	0.72	0.71	0.72	0.71	0.00	0.69	0.68	0.67	0.63	0.00	0.69	0.70	0.71	0.68
Lysine	1.18	1.16	1.17	1.20	0.00	1.15	1.11	1.10	1.07	0.00	1.14	1.18	1.17	1.15
Methionine	0.25	0.24	0.25	0.23	0.00	0.25	0.23	0.25	0.23	0.00	0.25	0.25	0.23	0.21
Valine	0.87	0.85	0.86	0.88	0.00	0.84	0.82	0.80	0.79	0.00	0.84	0.85	0.85	0.85
Isoleucine	0.84	0.82	0.83	0.87	0.00	0.82	0.79	0.77	0.77	0.00	0.81	0.82	0.83	0.83
Leucine	1.41	1.38	1.40	1.47	0.00	1.35	1.31	1.30	1.31	0.00	1.36	1.39	1.38	1.40
Phenylalanine	0.90	0.87	0.88	0.94	0.00	0.86	0.84	0.82	0.83	0.00	0.86	0.87	0.87	0.89
Tryptophan	0.25	0.24	0.26	0.23	0.00	0.26	0.26	0.25	0.26	0.00	0.25	0.24	0.25	0.24
Dispensable (g/100g)														
Cysteine	0.25	0.26	0.26	0.27	0.00	0.27	0.24	0.25	0.20	0.00	0.14	0.14	0.13	0.12
Serine	0.93	0.92	0.92	0.97	0.01	0.90	0.88	0.86	0.86	0.01	0.90	0.91	0.91	0.92
Glycine	0.78	0.76	0.76	0.79	0.01	0.74	0.73	0.70	0.70	0.01	0.75	0.76	0.74	0.75
Aspartic acid	2.14	2.11	2.13	2.26	0.00	2.09	2.00	1.98	2.03	0.00	2.06	2.11	2.12	2.16
Glutamic acid	3.35	3.30	3.34	3.62	0.00	3.26	3.12	3.09	3.23	0.00	3.23	3.31	3.31	3.46
Alanine	0.82	0.81	0.81	0.82	0.00	0.79	0.77	0.76	0.74	0.00	0.79	0.81	0.80	0.78
Proline	0.91	0.88	0.89	0.95	0.00	0.87	0.84	0.83	0.84	0.00	0.87	0.88	0.88	0.90
Tyrosine	0.58	0.62	0.59	0.62	0.00	0.56	0.56	0.53	0.55	0.00	0.58	0.57	0.59	0.59

1 SBM-A, -B, and -C are diets containing soybean meals derived from Manitoba (MB)-grown soybean; ON-SBM = diets containing soybean meal derived from Ontario-grown soybean.

2 N-free diet.

**Table 4:** Ingredient and nutrient composition of diets used in performance studies in pullets (wk-9 to -12, grower and wk-13 to -16, developer phases) and wk-59 to -64 laying hens (as-is basis).^1^

	Growers (wk-9 to 12)				Developers (wk-13 to 16)				Layers (wk-59 to 64)			
Ingredient (%)	SBM-A	SBM-B	SBM-C	ON-SBM	SBM-A	SBM-B	SBM-C	ON-SBM	SBM-A	SBM-B	SBM-C	ON-SBM
Corn	45.8	45.8	45.8	46.7	52.2	52.1	52.1	52.4	47.4	47.3	47.3	56.7
Wheat middling	21.0	21.0	21.0	20.1	16.2	16.4	16.4	16.1	10.0	10.0	10.0	0.6
SBM	28.0	28.0	28.0	28.0	26.0	26.0	26.0	26.0	28.5	28.5	28.5	28.5
Corn oil	-	-	-	-	-	-	-	-	0.33	0.33	0.33	0.37
Monocalcium phosphate	1.31	1.31	1.31	1.31	2.23	2.25	2.25	2.20	10.7	10.7	10.7	10.7
Vit-Min premix^2^	1.50	1.50	1.50	1.50	1.50	1.50	1.50	1.50	1.50	1.50	1.50	1.50
Limestone	1.96	1.98	1.98	1.92	1.37	1.37	1.37	1.37	1.16	1.16	1.16	1.22
Salt	0.44	0.44	0.44	0.44	0.44	0.44	0.44	0.44	0.44	0.44	0.44	0.45
*Calculated nutrient*												
ME (Poultry, kcal/kg)	3078	3080	3073	3053	3070	3073	3066	3046	2856	2859	2851	2854
CP	18.8	18.9	19.2	20.5	17.8	17.9	18.1	19.4	17.2	17.3	17.6	18.2
Fat	5.05	5.06	4.86	4.66	4.85	4.86	4.67	4.49	5.03	5.04	4.84	4.59
Crude fibre	3.38	3.33	3.22	3.33	3.05	3.01	2.90	3.04	2.57	2.52	2.41	2.00
Calcium	1.20	1.20	1.20	1.20	1.30	1.30	1.30	1.30	4.30	4.30	4.30	4.30
Total phosphorus	0.69	0.71	0.70	0.70	0.67	0.69	0.68	0.68	0.58	0.60	0.59	0.55
Av. phosphorus	0.45	0.45	0.45	0.45	0.45	0.45	0.45	0.45	0.40	0.40	0.40	0.40
Lysine	0.94	0.96	0.95	0.99	0.88	0.89	0.88	0.92	0.89	0.91	0.90	0.90
Methionine	0.28	0.29	0.29	0.28	0.27	0.28	0.27	0.27	0.27	0.27	0.27	0.26
Threonine	0.65	0.65	0.65	0.66	0.61	0.61	0.61	0.62	0.61	0.61	0.61	0.60
Tryptophan	0.22	0.22	0.22	0.22	0.20	0.20	0.20	0.20	0.19	0.20	0.20	0.18
Arginine	1.11	1.11	1.11	1.20	1.03	1.04	1.03	1.12	1.02	1.03	1.02	1.06
Sodium	0.20	0.20	0.20	0.20	0.20	0.20	0.20	0.20	0.20	0.20	0.20	0.20
Chloride	0.30	0.30	0.30	0.30	0.30	0.30	0.30	0.30	0.30	0.30	0.30	0.30
*Analyzed nutrient*												
DM	89.7	89.6	89.7	89.2	89.6	89.5	89.8	89.7	91.0	91.3	91.4	90.9
ME (Poultry, kcal/kg)	3144	3150	3132	3116	3120	3115	3129	3124	2919	2928	2960	2954
CP	19.3	19.2	19.2	20.6	18.3	18.1	18.6	20.0	16.7	17.5	18.2	18.5
Fat	2.92	2.81	2.85	2.63	2.98	2.75	2.88	2.80	2.24	2.08	2.32	2.16
Crude fibre	5.37	5.53	5.18	4.82	5.16	5.17	5.24	5.03	5.31	5.47	5.41	5.27
Calcium	0.99	1.14	1.06	1.02	1.14	1.08	1.11	1.20	5.24	5.07	5.00	4.71
Total Phosphorus	0.68	0.68	0.68	0.69	0.72	0.70	0.69	0.73	0.56	0.51	0.51	0.48

1 SBM-A, -B, and -C are diets containing SBM derived from Manitoba (MB)-grown soybean; ON-SBM = diets containing SBM derived from Ontario-grown soybean.

2 Vitamin-mineral premix supplied per kilogram of diet: 11,000 IU of vitamin A; 3,000 IU of vitamin D_3_, 20 IU of vitamin E, 3 mg of vitamin K_3_ (as menadione), 0.02 mg of vitamin B_12_, 0.2 mg of biotin, 6.5 mg of riboflavin, 4 mg of folic acid, 10 mg of calcium pantothenate, 39.9 mg of niacin, 2.2 mg of thiamine, 4.5 mg of pyridoxine, 1000 mg of choline chloride, 125 mg antioxidant (ethoxyquin), 66 mg of manganese oxide, 70 mg of zinc oxide, 80 mg of ferrous sulfate, 10 mg of copper sulfate, 0.3 mg of sodium selenite, 0.4 mg of calcium iodate, 0.67 mg of sodium chloride (salt).

**Table 5:** Analyzed amino acid contents of diets used in performance studies in pullets (wk-9 to -12, grower and wk-13 to -16, developer phases) and wk-59 to -64 laying hens (as-is basis).^1^

	Growers (wk-9–12)				Developers (wk-13–16)				Layers (wk-59–64)			
Amino acids	SBM-A	SBM-B	SBM-C	ON-SBM	SBM-A	SBM-B	SBM-C	ON-SBM	SBM-A	SBM-B	SBM-C	ON-SBM
Indispensable (g/100g)												
Arginine	1.18	1.19	1.19	1.33	1.12	1.09	1.19	1.27	1.08	1.02	1.11	1.15
Histidine	0.57	0.55	0.52	0.58	0.51	0.48	0.52	0.55	0.48	0.45	0.47	0.51
Threonine	0.74	0.73	0.74	0.76	0.71	0.68	0.75	0.74	0.67	0.65	0.69	0.68
Lysine	1.00	1.02	1.01	1.04	0.96	0.93	1.02	1.02	0.92	0.89	0.96	0.97
Methionine	0.30	0.30	0.30	0.32	0.30	0.28	0.31	0.31	0.27	0.28	0.28	0.29
Valine	0.92	0.92	0.92	0.96	0.88	0.85	0.92	0.94	0.84	0.81	0.86	0.86
Isoleucine	0.81	0.81	0.82	0.85	0.76	0.74	0.82	0.83	0.74	0.72	0.76	0.78
Leucine	1.67	1.65	1.66	1.74	1.62	1.56	1.69	1.74	1.55	1.51	1.58	1.64
Phenylalanine	0.94	0.94	0.94	1.00	0.90	0.86	0.95	0.98	0.86	0.83	0.87	0.91
Tryptophan	0.22	0.23	0.24	0.25	0.23	0.22	0.24	0.25	0.20	0.21	0.21	0.22
Dispensable (g/100g)												
Cysteine	0.32	0.32	0.33	0.33	0.32	0.32	0.35	0.34	0.29	0.30	0.28	0.28
Serine	0.99	0.98	0.99	1.04	0.93	0.90	0.99	1.01	0.90	0.88	0.92	0.92
Glycine	0.82	0.82	0.82	0.87	0.79	0.76	0.82	0.84	0.73	0.70	0.74	0.73
Aspartic acid	1.85	1.83	1.86	1.97	1.76	1.70	1.87	1.93	1.71	1.66	1.77	1.89
Glutamic acid	3.67	3.63	3.67	3.88	3.40	3.30	3.55	3.74	3.23	3.15	3.30	3.40
Alanine	0.99	0.98	1.01	1.01	0.97	0.94	1.01	1.02	0.92	0.90	0.92	0.94
Proline	1.21	1.20	1.21	1.25	1.14	1.11	1.18	1.23	1.08	1.05	1.09	1.12
Tyrosine	0.68	0.65	0.68	0.72	0.65	0.63	0.69	0.71	0.65	0.63	0.66	0.65

1 SBM-A, -B, and -C are diets containing soybean meals derived from Manitoba (MB)-grown soybean; ON-SBM = diets containing soybean meal derived from Ontario-grown soybean.

### Digestibility Assays

Ileal endogenous losses of CP and AA during the grower (12-wk-old) and the developer (16-wk-old) phases are shown in Table 6. The ileal endogenous loss of CP was higher (*P* = 0.016) for birds in the grower compared to the developer phase, possibly a result of increased secretion of digestive enzymes, rate of digestion and absorption of endogenous protein with older than younger birds (Nasset, 1972; Al-Qahtani et al., 2021). On the other hand, ileal endogenous losses of individual AA were similar (*P >* 0*.*05) in birds at the grower and developer stages, except for histidine, which was higher (*P* = 0.033) in the former than the latter stage. The values for the ileal endogenous AA losses observed in this study conform with published data for 33 to35 day-old broilers (Ravindran et al., 2004), for 21 d-old broilers and 30- or 50-wk-old laying hens (Adedokun et al., 2014) and the mean value derived from several previous studies in broiler chickens (Adeola et al., 2016).

**Table 6:** Ileal endogenous CP and amino acid losses in pullets (grower, wk-12 and developer, wk-16 of age).^1^

Amino acids (g/kg DMI)	Grower (wk-12-old)	Developer (wk-16-old)	SEM	*P*-value
CP^2^	27.7^a^	16.3^b^	2.79	0.016
Indispensable				
Arginine	0.325	0.321	0.056	0.959
Histidine	0.227^a^	0.143^b^	0.024	0.033
Threonine	0.610	0.646	0.083	0.766
Lysine	0.375	0.337	0.067	0.694
Methionine	0.110	0.095	0.023	0.651
Valine	0.446	0.472	0.068	0.796
Isoleucine	0.307	0.336	0.055	0.715
Leucine	0.532	0.500	0.090	0.803
Phenylalanine	0.277	0.305	0.050	0.698
Tryptophan	0.128	0.111	0.018	0.507
Mean (Indispensable)	**0.335**	**0.327**		
Dispensable				
Cysteine	0.242	0.245	0.027	0.925
Serine	0.589	0.628	0.080	0.738
Glycine	0.439	0.431	0.055	0.916
Aspartic acid	0.729	0.671	0.106	0.710
Glutamic acid	0.956	0.829	0.138	0.533
Alanine	0.405	0.322	0.056	0.318
Proline	0.530	0.605	0.070	0.467
Tyrosine	0.228	0.284	0.043	0.383
Mean (Dispensable)	**0.494**	**0.482**		

1 Means followed by different letters within rows differ (*P* < 0.05). *n* = 6 (per replicate cage).

The SID values for CP and AA in the 4 test SBM during the grower (12-wk-old), developer (16-wk-old) and layer (44-wk-old) phases are summarized in Table 7. In the pullet phase (grower and developer), most SID coefficients of AA among the 4 sources of SBM were not significantly different. However, in the grower phase lower (*P* < 0.05) SID value for cysteine was evident for SBM-C (but not the case for SBM-A or SBM-B) compared to the ON meal, and in the developer phase the SID value for methionine, phenylalanine, and cysteine were on average 3.9, 2.2, and 1.5% units higher in SBM-A, SBM-B, and SBM-C, respectively, compared to the ON meal. Notably, the difference in the SID values between the SBM were primarily the S-containing AA. The reason for the differences among SBM samples could be because in soybeans methionine and cysteine are the first limiting AA (Sexton et al., 1998; Krishnan and Jez, 2018), yet, the relatively low content of these AA in the composition of the SBM-containing diets may result in insufficient or imbalanced AA supply which may alter metabolic utilization of these AA (Kong and Adeola, 2013; Sung et al., 2023). Moreover, the grower diets relative to the developer diets, usually demands a nutrient dense diet composition, including AA (NRC, 1994). Alternatively, although all diets in each phase were formulated to contain similar CP content, it could be possible that dietary protein/AA may not be completely digested or utilized by birds because of the presence of some indigestible fractions or antinutritional components in the diet (Beski et al., 2015). In a separate study, conducted under the same project utilizing the same SBM samples (Pinar, 2023), it was observed that SBM-C contained higher (170.9 g/kg) content of non-starch polysaccharides (**NSP**) compared to the SBM-A, SBM-B or the ON-SBM (167.6, 164.6, or 143.2 g/kg, respectively). In addition, oligosaccharides (raffinose family) content for the MB grown soybean, on average, was 74.9 g/kg compared to 57.4 g/kg for the ON meal. In soybean meal, NSP and oligosaccharides contribute to the indigestible fraction of carbohydrates (Choct et al., 2010), hence poorly digested in poultry, particularly in younger birds (Almirall et al., 1995). Moreover, the soybean's raffinose content negatively correlates with the ileal digestibility of AA (Li et al., 2017). Hence, in the grower phase, the lower SID values of cysteine for the SBM derived from MB-grown soybean (SBM-C) compared to that in the ON meal may relate the presence of higher antinutritional contents. It is generally accepted that the digestive capacity of birds continues to develop with maturity (Morel et al., 2006), hence, the differences in the coefficient of SID of AA, noted between the grower and the developer phases may relate to differences in physiological status, enzyme secretions (Ravindran et al., 2017). Although the SID coefficients of AA of feed ingredients are generally rare in pullets, making direct comparison between previous studies challenging, the results of the present study are consistent with previous published date using SBM in broiler chickens and mature laying hens (Adedokun et al., 2014), growing pigs (Li et al., 2017), and in general, poultry (Evonik, 2016). For instance, considering the 5 key AA in poultry diets, the average SID values of the expeller-pressed MB grown meal in the grower phase (90.1 ± 1.72, 91.8 ± 1.74, 85.4 ± 2.76, 87.2 ± 1.25 and 81.9 ± 1.98%) and developer phase (87.8 ± 1.72, 90.2 ± 1.74, 83.7 ± 2.76, 87.9 ± 2.31 and 81.7 ± 2.27%) observed in this study, were similar to those for expeller SBM in poultry (91, 90, 85, 89, and 82%) published database service by Evonik (2016) for methionine, lysine, threonine, tryptophan, and cysteine, respectively. Similarly, average SID values for 3 categories of SBM for 21 d-old broiler chickens (87.8 ± 0.80, 88.6 ± 0.46, 82.5 ± 0.89, and 77.3 ± 1.28%) and for 30- and 50 wk-old laying hens (91.8 ± 0.99, 90.1 ± 1.07, 87.2 ± 0.68, and 85.7 ± 1.37%) reported by Adedokun et al. (2014) for methionine, lysine, threonine, and cysteine, respectively, are similar to those observed in the pullet phase, herein, in the current study.

**Table 7:** Standardized ileal digestibility of CP and amino acids in 12-wk-old grower pullets, 16-wk-old developer pullets and 44-wk-old laying hens fed expeller-pressed soybean meal.^1^

	Growers (wk-12-old)					Developers (wk-16-old)					Layers (wk-44-old)				
Components (%)	SBM-A	SBM-B	SBM-C	ON-SBM	SEM	SBM-A	SBM-B	SBM-C	ON-SBM	SEM	SBM-A	SBM-B	SBM-C	ON-SBM	SEM
CP	89.9	91.1	89.1	90.6	0.91	87.4	90.5	87.1	86.4	1.17	81.7^a^	76.9^ab^	78.6^ab^	66.3^b^	3.49
Indispensable Amino Acids															
Arginine	94.8	95.4	94.1	95.5	0.68	93.2	94.5	92.7	91.6	0.66	83.4	82.0	83.5	79.7	3.15
Histidine	88.1	90.2	88.2	90.1	0.97	90.7	93.3	90.9	91.3	0.88	87.9	85.5	85.9	81.7	2.20
Threonine	85.2	87.0	84.0	86.2	1.19	82.8	86.8	81.5	80.4	1.73	66.2	62.8	64.8	49.8	4.95
Lysine	91.7	92.7	91.1	93.2	0.96	89.4	92.2	89.0	88.2	1.03	76.7	75.3	76.7	69.0	3.95
Methionine	90.3	90.6	89.3	91.2	1.66	87.1^ab^	89.8^a^	86.6^ab^	83.9^b^	1.37	69.3	60.8	65.6	53.7	6.82
Valine	89.0	90.2	87.7	90.1	1.00	86.8	89.7	85.8	84.9	1.27	73.0	70.1	72.7	61.6	4.31
Isoleucine	91.0	91.8	89.7	91.7	0.87	88.5	91.0	87.6	86.4	1.13	76.4	73.2	76.1	66.3	3.84
Leucine	90.3	91.2	89.3	91.3	0.91	88.8	91.3	88.2	87.1	1.05	76.7	74.1	76.4	66.2	3.93
Phenylalanine	92.5	93.4	91.8	93.5	0.76	90.5^ab^	92.6^a^	89.9^ab^	88.8^b^	0.91	79.5	77.1	79.1	71.0	3.56
Tryptophan	86.8	88.6	86.2	87.7	1.09	87.0	90.6	86.3	86.5	1.16	75.4	68.8	75.5	61.7	4.38
Dispensable Amino acids															
Cysteine	81.5^ab^	84.0^ab^	80.1^b^	84.7^a^	1.13	82.1^ab^	83.8^a^	79.3^ab^	75.8^b^	1.80	67.1	49.1	52.8	35.0	7.58
Serine	88.2	89.6	87.0	89.6	0.86	86.7	89.5	86.0	85.4	1.16	73.2	70.7	72.2	61.9	4.00
Glycine	85.3	87.0	84.1	87.7	1.02	83.8	87.8	83.1	82.9	1.41	70.5	67.7	70.6	57.4	4.29
Aspartic acid	88.9	90.1	87.9	90.4	0.77	87.8	90.2	86.7	86.5	1.14	77.6	74.9	76.5	67.5	3.29
Glutamic acid	92.7	93.5	91.8	93.3	0.64	91.8	93.5	90.9	90.2	0.80	83.0	80.8	81.9	75.4	3.00
Alanine	88.7	90.0	87.6	89.7	1.06	88.0	90.9	87.0	85.9	1.26	74.2	69.8	71.5	57.5	4.55
Proline	89.3	90.4	88.2	90.4	0.77	87.6	90.2	86.7	86.2	1.13	76.9	74.7	76.1	67.6	3.41
Tyrosine	92.0	93.2	90.9	92.8	0.92	87.7	90.6	86.1	85.9	1.25	72.2	66.4	72.8	63.3	5.35

1 SBM-A, -B, and -C are diets containing soybean meals derived from Manitoba (MB)-grown soybean; ON-SBM = diets containing soybean meal derived from Ontario-grown soybean. Different superscripts in a row within a phase are significantly different at *P* < 0.05. *n* = 6 for all treatments except in the layer phase, where *n* = 5 (per replicate cage).

In the layer phase (44-wk-old birds), no differences in SID coefficients of CP and AA were noted between the SBM types obtained from MB compared with that from ON (Table 7). However, notable differences were observed between the SID values of CP and AA of SBM in layer and the pullet phases (grower and developer), being lower (*P* < 0.0001) for the former class of birds than for the latter (Supplementary data, Table S1). These differences may, in part, be attributed to differences in age, physiological status, nutritional adequacy of test diets (Huang et al., 2006; Qiu et al., 2023) and differences in bacterial communities in the gut (Guo et al., 2018; Neijat et al., 2019). Although SID coefficients of AA of feed ingredients in egg-laying hens are rare, comparisons based on data for solvent-extracted SBM (CP content in the range of 45–48%), fed to laying hens aged 30- and 50-wk-old (Adedokun et al., 2014), the SID values of most AA were higher than that obtained in the current study. Generally, SBM with higher CP, have been shown to contain lower fiber content (Medic et al., 2014; Thakur and Hurburgh, 2007). In this study, adding cellulose to both N-free and experimental diets, as done in previous digestibility assays for growing pigs (Kaewtapee et al., 2018) as a means to adjust energy values, likely elevated the fiber content in diets. Cellulose contributes to the undigested components in the terminal ileum (Khalil et al., 2022), hence may be linked to an increased loss of endogenous ileal AA that may have influenced the SID values of AA in the layer phase compared to the pullet phase. Hence, the high fiber content in the diets in the current study, particularly in the laying hen diets compared to the pullet phase, could potentially have decreased the digestibility coefficients of AA in the SBM (Dilger et al., 2004). This result aligns with a previous study (Neijat et al., 2019) showing that fiber-degrading microbial communities, primarily from the phyla Bacteroidetes and Firmicutes, are more dominant in the gut for pullets compared with the layers. Although, the inclusion of cellulose in this study, was within limits (3–5% of diet) that may not cause any negative effects in nutrient digestibility or growth performance of different poultry species (Cao et al., 2003; Jiménez-Moreno et al., 2009), a separate assessment of endogenous ileal AA flow in the layer phase was required.

In general, in both the pullet and layer phases, cysteine was least digestible in all phases for all types of the SBM in this study and supported by previous reports (Ravindran et al., 2014; Evonik, 2016). Cysteine, like lysine (Karr-Lilienthal et al., 2006; Pahm et al., 2008), is affected by processing conditions. Factors such as temperature, pressure, and fat removal can affect cysteine concentrations (Wang and Parsons, 1998) possibly due to the formation of cross-linked sulfur AA like lysinoalanine or lanthionine (Pfeuti et al., 2019). Moreover, in the present study, sampling the entire ileum, that is, Meckel's diverticulum to the end of ileo-cecal junction, could contributed to a source of error in underestimating some digestibility values. Nonetheless, in the current study, the digestibility coefficients of CP and most AA for the expeller-pressed SBM, particularly in the grower and developer phases, were comparable or even higher than for solvent-extracted SBM (Hemetsberger et al., 2021; Marty and Chavez, 1993; Woodworth et al., 2001). For instance, in the grower phase, the SID values for most indispensable AA, particularly methionine (90.0 vs. 91.2%), cysteine (81.9 vs*.* 84.7%), lysine (91.8 vs. 93.2%), tryptophan (87.2 vs. 87.7%) and threonine (85.4 vs*.* 86.2%) on average for MB- vs*.* ON-SBM were higher than for solvent-extracted SBM (90, 79, 89, 89, and 83%, respectively) in broiler chickens (Evonik, 2016). In another study by Siegert et al. (2023), the medians of digestibility of the first-limiting AA for 18 solvent-extracted SBM samples from different origins in cecectomized laying hens were 91% for methionine, 78% for cysteine, 90% for lysine, 84% for threonine, and 88% for valine, slightly lower than those herein presented. Considering that the SBM products fed in the current study were not dehulled, it is likely that dietary fiber as a functional component of normal digestive organ, could play a role in stimulate gut health (Singh and Kim, 2021), increase feed retention time, and enhance nutrient digestibility (Shuaib et al., 2023).

The digestible (SID) contents of AA (Table 8) in the SBM also followed a similar trend as the SID coefficients of AA in the SBM types in all classes of chickens. In the grower phase, the digestible contents of the indispensable AA (Table 8) for SBM derived from MB-grown soybean were lower for most AA except for methionine, histidine, cysteine and methionine + cysteine, which were comparable to the ON-SMB. However, in the developer phase, the sum or the individual S-AA were lower for the ON meal compared to the MB-SBM. In addition, the digestible content of all AA in the layer phase were similar (*P >* 0*.*05) among the different types of SBM. These results are in agreement with previous reports (Ravindran and Bryden, 1999; Adedokun et al., 2008) that age/physiological status of the birds has a substantial impact on AA digestibility. Moreover, the values of digestible content of AA are most widely used to formulate poultry diets (Adeola et al., 2016; Ravindran et al., 2014), hence, necessitate age-specific nutrient digestibility values for efficient utilization of the MB-grown SBM.

**Table 8:** Standardized ileal digestible amino acid contents of expeller-pressed soybean meals grown in Manitoba vs*.* Ontario.^1^

	Growers (wk-12-old)					Developers (wk-16-old)					Layers (wk-44-old)				
Amino acids^2^	SBM-A	SBM-B	SBM-C	ON-Diet	SEM	SBM-A	SBM-B	SBM-C	ON-SBM	SEM	SBM-A	SBM-B	SBM-C	ON-SBM	SEM
Indispensable Amino Acids (%)															
Arginine	2.56^b^	2.60^b^	2.55^b^	2.93^a^	0.019	2.52^b^	2.58^b^	2.51^b^	2.81^a^	0.019	2.25	2.24	2.26	2.45	0.086
Histidine	1.03	1.04	1.01	1.04	0.011	1.06	1.07	1.05	1.05	0.010	1.03	0.98	0.99	0.94	0.024
Threonine	1.33^ab^	1.37^ab^	1.32^b^	1.40^a^	0.019	1.29	1.36	1.28	1.30	0.028	1.03	0.99	1.02	0.81	0.078
Lysine	2.37^b^	2.43^b^	2.37^b^	2.55^a^	0.025	2.31^b^	2.42^a^	2.32^ab^	2.42^a^	0.028	1.98	1.97	1.99	1.89	0.103
Methionine	0.55	0.58	0.55	0.56	0.010	0.53^b^	0.58^a^	0.54^b^	0.51^b^	0.008	0.42	0.39	0.41	0.33	0.043
Valine	1.68^b^	1.70^b^	1.66^b^	1.80^a^	0.019	1.64	1.69	1.62	1.70	0.025	1.38	1.32	1.37	1.23	0.081
Isoleucine	1.65^b^	1.64^b^	1.63^b^	1.81^a^	0.016	1.60^b^	1.63^ab^	1.59^b^	1.70^a^	0.021	1.38	1.31	1.39	1.31	0.069
Leucine	2.73^b^	2.76^b^	2.69^b^	3.00^a^	0.028	2.68^b^	2.77^ab^	2.66^b^	2.87^a^	0.033	2.32	2.24	2.30	2.18	0.119
Phenylalanine	1.79^b^	1.81^b^	1.77^b^	1.97^a^	0.015	1.75^b^	1.78^b^	1.74^b^	1.87^a^	0.019	1.53	1.50	1.53	1.50	0.069
Tryptophan	0.45^b^	0.48^a^	0.47^ab^	0.47^ab^	0.006	0.45^b^	0.49^a^	0.47^ab^	0.47^ab^	0.006	0.39	0.37	0.41	0.33	0.023
Dispensable Amino acids (%)															
Cysteine	0.47	0.49	0.47	0.47	0.007	0.48^a^	0.49^a^	0.46^ab^	0.42^b^	0.010	0.39^a^	0.29^ab^	0.31^ab^	0.18^b^	0.040
Serine	1.75^b^	1.77^b^	1.71^b^	1.91^a^	0.017	1.72^b^	1.77^ab^	1.69^b^	1.82^a^	0.024	1.45	1.40	1.42	1.32	0.074
Glycine	1.38^b^	1.40^b^	1.37^b^	1.53^a^	0.017	1.36	1.41	1.35	1.44	0.024	1.14	1.09	1.15	1.00	0.069
Aspartic acid	4.06^b^	4.08^b^	3.97^b^	4.48^a^	0.035	4.00^b^	4.08^ab^	3.92^b^	4.29^a^	0.054	3.54	3.39	3.46	3.35	0.149
Glutamic acid	6.55^b^	6.27^c^	6.45^b^	7.45^a^	0.045	6.49	6.26	6.38	7.20	0.060	5.87	5.42	5.75	6.02	0.207
Alanine	1.54^b^	1.57^b^	1.54^b^	1.67^a^	0.019	1.53	1.58	1.53	1.60	0.023	1.29	1.21	1.26	1.07	0.079
Proline	1.71^b^	1.74^b^	1.71^b^	1.94^a^	0.015	1.68^b^	1.73^b^	1.68^b^	1.85^a^	0.023	1.48	1.43	1.48	1.45	0.066
Tyrosine	1.29^b^	1.24^b^	1.26^b^	1.39^a^	0.013	1.23^ab^	1.20^b^	1.19^b^	1.29^a^	0.018	1.01	0.88	1.00	0.95	0.073
ΣSAA^2^	1.02	1.08	1.02	1.03	0.016	1.01ab	1.06a	1.00ab	0.94b	0.018	0.81	0.67	0.71	0.51	0.075
ΣAAA^3^	3.08^b^	3.05^b^	3.03^b^	3.37^a^	0.027	2.97^b^	3.00^b^	2.92^b^	3.16^a^	0.036	2.55	2.38	2.53	2.45	0.141

1 SBM-A, -B, and -C are diets containing soybean meals derived from Manitoba (MB)-grown soybean; ON-SBM = diets containing soybean meal derived from Ontario-grown soybean. Different superscripts in a row within a phase are significantly different at *P* < 0.05. *n* = 6 for all treatments except in the layer phase, where *n* = 5 (per replicate cage).

2 Sum of sulfur-containing amino acids (Methionine + Cysteine).

3 Sum of aromatic amino acids (Phenylalanine + Tyrosine).

### Performance Assays

The performance of the pullets (wk-9– wk-16) and laying hens (wk-59– wk-64) are presented in Table 9, Table 10, respectively. The diets containing each of the 4 SBM types did not result in any significant effect in pullets (grower phase, wk-9 to wk-12, and developer phase wk-13 to wk-16), performance with respect to feed intake, BW gain, and FCR (Table 9). Similarly, in the layer phase, the source of SBM did not affect (*P >* 0.05) feed intake, egg production, egg weight, egg mass and FCR (Table 10). In the layer stage, although feed consumption met the breed guide recommendation for Dekalb White (Commercial CS management guide, North American Version L2221-1), birds fed diets containing ON-SBM had lower (*P* < 0.05) intake (104 g/bird/day) in the last week (wk-64-old birds) compared to those fed diets containing MB-SBM (on average, 110 g/bird/day). However, this difference in feed consumption did not influence BW gain or FCR between both groups of birds. In the current study, all diets were formulated based on a similar inclusion level of each SBM type, that is, 28, 26 and 29% of diet in the grower, developer, and layer phases, respectively, consequently resulting in higher CP content for the ON-SBM-containing diet compared to those containing the MB meals. Nonetheless, in laying hens, egg production was not affected by the type of SBM. However, there was a tendency of g feed intake/g egg mass for ON-SBM to be greater than that for Manitoba SBM (Table 8). The acquisition of SBM samples for the whole study was delayed due to COVID-19 restrictions, disrupting the timeline for both digestibility and performance experiments. Thus, regretfully, the present study did not utilize the SID of AA values to formulate the diets in the performance experiment. There are variations in the standardized ileal digestible values of AA among the SBM samples within a region and between regions, as demonstrated in the current study and in previous studies (Ravindran et al., 2014; Siegert et al., 2023). Hence, accurate feed formulation should consider digestible AA content. In addition, supplementing diets with crystalline AA can address variations in standardized ileal digestible AA content among SBM samples under practical /commercial conditions. However, overall, the comparable performance outcome (growth/production variables) of birds among the different test SBM, herein demonstrated in the current study, may be attributed to the high-quality AA profile of the SBM derived from MB-grown soybean. Egg components, that is, egg white, yolk and shell, as well as shell thickness, were not influenced by the SBM types, however, there was an age effect (*P <* 0.05) on egg quality (eggshell weight and thickness), whereby eggshell quality increased with age of the birds but no interaction effects between age and treatment was noted (Table 11).

**Table 9:** Feed intake and FCR in pullets (Grower, wk-9 to 12 and Developer, wk-13 to 16).^1^

Age (wk)	SBM-A	SBM-B	SBM-C	ON-SBM	SEM	*P*-value
Feed Consumption (g/bird/d)						
Grower phase						
9	56.4	56.6	55.8	55.8	0.54	0.615
10	56.8	56.6	55.8	56.2	0.52	0.488
11	59.1	59.6	58.8	59.2	0.62	0.863
12	60.6	61.0	60.5	60.6	0.62	0.925
Developer phase						
13	67.0	68.2	68.3	67.1	0.75	0.488
14	66.2	66.4	66.2	65.7	0.69	0.902
15	65.4	65.8	65.9	65.4	0.71	0.927
16	67.6	67.9	66.8	68.2	0.78	0.621
FCR (g FI/wk: g BWG/wk) per bird						
Grower phase						
9	3.73	3.69	3.74	3.69	0.06	0.895
10	4.67	4.53	4.49	4.54	0.10	0.634
11	5.11	5.28	5.25	5.04	0.12	0.410
12	5.91	5.98	5.98	5.91	0.13	0.962
Developer phase						
13	6.46	6.13	6.46	6.12	0.12	0.053
14	7.79	7.45	7.28	7.42	0.22	0.434
15	8.11	8.19	8.36	8.95	0.36	0.372
16	7.04	7.61	7.26	7.17	0.35	0.679

1 SBM-A, -B, and -C are diets containing soybean meals derived from Manitoba (MB)-grown soybean; ON-SBM = diets containing soybean meal derived from Ontario-grown soybean. Different superscripts within a row are significantly different at *P*<0.05. *n* = 5 (per replicate cage).

**Table 10:** Performance of laying hens (wk-59 to -64 of age).^1^

Age (wk)	SBM-A	SBM-B	SBM-C	ON-SBM	SEM	*P*-value
Feed Consumption (g/bird/d)						
59	90.0	89.7	89.3	86.7	1.15	0.215
60	99.7	96.6	98.5	96.9	1.42	0.410
61	100.9	100.4	102.1	98.9	1.11	0.249
62	110.1	108.8	109.7	105.2	1.36	0.074
63	109.2	109.3	108.4	105.9	1.42	0.356
64	112.5^a^	112.5^a^	111.8^ab^	108.1^b^	1.01	0.020
Egg production (% Hen-day egg-lay)						
59	92.4	88.8	91.0	92.0	1.60	0.405
60	94.4	91.6	93.7	92.0	1.50	0.493
61	93.5	90.9	91.0	92.1	1.56	0.628
62	98.4	97.4	95.4	97.0	1.43	0.532
63	93.6	92.7	94.2	94.4	0.91	0.543
64	96.1	92.7	95.5	94.5	2.19	0.707
Egg weight (g/egg)						
59	61.5	60.6	60.8	60.6	0.51	0.564
60	60.5	60.5	60.5	60.7	0.35	0.939
61	60.6	60.3	60.8	61.0	0.41	0.669
62	61.4	61.1	61.1	61.2	0.41	0.957
63	61.7	61.5	61.5	61.3	0.40	0.934
64	62.1	61.9	61.4	61.8	0.40	0.710
Egg mass (g/egg)						
59	56.8	53.8	55.4	55.7	0.93	0.186
60	57.1	55.4	56.7	55.9	0.85	0.484
61	56.6	54.8	55.3	56.2	1.03	0.615
62	60.4	59.6	58.3	59.3	0.95	0.492
63	57.7	57.0	57.9	57.9	0.64	0.714
64	59.6	57.4	58.7	58.3	1.36	0.711
FCR (g feed intake/g egg mass)						
59	1.59	1.67	1.61	1.56	0.036	0.175
60	1.75	1.74	1.71	1.73	0.019	0.555
61	1.81	1.83	1.85	1.76	0.026	0.128
62	1.82	1.83	1.89	1.77	0.032	0.138
63	1.89	1.92	1.87	1.82	0.024	0.067
64	1.89	1.97	1.91	1.85	0.046	0.362

1 SBM-A, -B, and -C are diets containing soybean meals derived from Manitoba (MB)-grown soybean; ON-SBM = diets containing soybean meal derived from Ontario-grown soybean. Different superscripts within a row are significantly different at *P* < 0.05. *n* = 20 (per replicate cage).

**Table 11:** Egg quality measures in laying hens (wk-62 to wk-64 of hen age).^1^

	Egg wt.	Egg yolk wt.	Eggshell wt.	Egg white wt.	Shell thickness
Treatment	(g/egg)	(g/egg)	(g/egg)	(g/egg)	(mm)
SBM-A	61.2	16.9	5.98	38.4	0.42
SBM-B	61.3	16.9	5.84	38.5	0.42
SBM-C	61.8	17.0	5.98	38.7	0.43
ON-SBM	61.0	16.9	5.95	37.9	0.43
SEM	0.43	0.13	0.05	0.36	0.003
**Age (wk)**					
62	61.5	16.8^b^	5.89^b^	38.7	0.41^b^
63	60.9	16.8^b^	5.92^ab^	38.2	0.42^b^
64	61.5	17.1^a^	6.02^a^	38.3	0.45^a^
SEM	0.28	0.09	0.04	0.24	0.002
*P*-value					
Treatment	0.563	0.949	0.159	0.494	0.057
Age	0.085	0.008	0.013	0.161	<.0001
Treatment*Age	0.810	0.344	0.745	0.581	0.133

1 SBM-A, -B, and -C are diets containing soybean meals derived from Manitoba (MB)-grown soybean; ON-SBM = diets containing soybean meal derived from Ontario-grown soybean. Different superscripts within a column (variable) are significantly different at *P* < 0.05. *n* = 20 (per replicate cage).

In summary, the results from this study confirm that high variability in protein content exists between different sources of soybean meal obtained from within a region and between different regions. Despite the low CP of the soybean meal derived from MB-grown soybean, the coefficient of SID of most AA were comparable with the ON-soybean meal, herein, evaluated in pullets and laying hens. Moreover, growth/production performance including feed intake, BW gain and FCR in pullets, and egg production and quality in layers were similar between the different sources of soybean meal. These outcomes may be linked to the higher values of key essential AA per unit of protein content in the MB-soybean meal *vs.* the ON-soybean meal for poultry nutrition. Hence, the findings of this study support the potential use of soybean meal derived from MB-grown soybean as a feed ingredient in egg-producing hens. Moreover, the data, herein determined, provides age-specific datasets of the SID content of AA to optimize dietary formulations for efficient utilization of the soybean grown in Western Canada. Furthermore, the impact of the study contributes to enhancing processing capacity in the region, for a sustainable utilization of the locally produced soybean as a potential protein source for poultry.

## DISCLOSURES

The authors declare no conflicts of interest.

## Appendix Supplementary materials

Image, application 1

## Footnotes

Supplementary material associated with this article can be found in the online version at doi:10.1016/j.psj.2024.104081.
