# Supplementary material for: Standardized ileal amino acid digestibility and performance indices in pullets and laying hens fed expeller-pressed Canadian prairie soybean meal
Source: Poult Sci. 2024 Jul 10;103(10):104081. doi: 10.1016/j.psj.2024.104081 (PMC11337718; doi:10.1016/j.psj.2024.104081)
Supplement: Supplementary file 1 [file mmc1.docx]

**TABLE S1:** Comparison of treatment (SBM type), phase and treatment × phase effect on standardized ileal digestibility of 5 key amino acids analysed for Manitoba-grown SBM^1^

|  |  |  | **Amino acids (%)** | | | | |
| --- | --- | --- | --- | --- | --- | --- | --- |
| **Phase** | **Treatment** |  | **Methionine** | **Cysteine** | **Lysine** | **Threonine** | **Tryptophan** |
| Grower | SBM-A |  | 90.3^a^ | 81.5^a^ | 91.7^a^ | 85.2^a^ | 86.8^a^ |
| Grower | SBM-B |  | 90.6^a^ | 84.0^a^ | 92.7^a^ | 87.0^a^ | 88.6^a^ |
| Grower | SBM-C |  | 89.3^a^ | 80.1^a^ | 91.1^a^ | 84.0^a^ | 86.2^a^ |
| Developer | SBM-A |  | 87.1^a^ | 82.1^a^ | 89.4^a^ | 82.8^a^ | 87.0^a^ |
| Developer | SBM-B |  | 89.8^a^ | 83.8^a^ | 92.2^a^ | 86.8^a^ | 90.6^a^ |
| Developer | SBM-C |  | 86.6^a^ | 79.3^a^ | 89.0^a^ | 81.5^a^ | 86.3^a^ |
| Layer | SBM-A |  | 69.3^b^ | 67.1^b^ | 76.7^b^ | 66.2^b^ | 75.4^b^ |
| Layer | SBM-B |  | 60.8^b^ | 49.1^b^ | 75.3^b^ | 62.8^b^ | 68.8^b^ |
| Layer | SBM-C |  | 65.6^b^ | 52.8^b^ | 76.7^b^ | 64.8^b^ | 75.5^b^ |
|  | ***P*-values** |  |  |  |  |  |  |
|  | Treatment |  | 0.789 | 0.106 | 0.372 | 0.270 | 0.684 |
|  | Phase |  | <.0001 | <.0001 | <.0001 | <.0001 | <.0001 |
|  | Phase × Treatment |  | 0.513 | 0.052 | 0.528 | 0.362 | 0.062 |
|  |  |  |  |  |  |  |  |
| **Differences (*P*-values) of Phase effects** | | | | | | | |
| ***Grower vs. Layer phase*** | |  |  |  |  |  |  |
| **Grower** | **Layer** |  |  |  |  |  |  |
| SBM-A | SBM-A |  | 0.0008 | 0.0514 | <.0001 | <.0001 | 0.0123 |
| SBM-A | SBM-B |  | <.0001 | <.0001 | <.0001 | <.0001 | <.0001 |
| SBM-A | SBM-C |  | 0.0001 | <.0001 | <.0001 | <.0001 | 0.0138 |
| SBM-B | SBM-A |  | 0.0006 | 0.0104 | <.0001 | <.0001 | 0.0019 |
| SBM-B | SBM-B |  | <.0001 | <.0001 | <.0001 | <.0001 | <.0001 |
| SBM-B | SBM-C |  | <.0001 | <.0001 | <.0001 | <.0001 | 0.0022 |
| SBM-C | SBM-A |  | 0.0016 | 0.114 | <.0001 | 0.0002 | 0.0218 |
| SBM-C | SBM-B |  | <.0001 | <.0001 | <.0001 | <.0001 | 0.0001 |
| SBM-C | SBM-C |  | 0.0002 | <.0001 | <.0001 | <.0001 | 0.0242 |
| ***Developer vs. Layer phase*** | |  |  |  |  |  |  |
| **Developer** | **Layer** |  |  |  |  |  |  |
| SBM-A | SBM-A |  | 0.0076 | 0.0351 | 0.0007 | 0.0007 | 0.0102 |
| SBM-A | SBM-B |  | <.0001 | <.0001 | 0.0002 | <.0001 | <.0001 |
| SBM-A | SBM-C |  | 0.0011 | <.0001 | 0.0007 | 0.0003 | 0.0115 |
| SBM-B | SBM-A |  | 0.0011 | 0.0118 | <.0001 | <.0001 | 0.0002 |
| SBM-B | SBM-B |  | <.0001 | <.0001 | <.0001 | <.0001 | <.0001 |
| SBM-B | SBM-C |  | 0.0002 | <.0001 | <.0001 | <.0001 | 0.0002 |
| SBM-C | SBM-A |  | 0.0112 | 0.1711 | 0.001 | 0.0024 | 0.0214 |
| SBM-C | SBM-B |  | 0.0001 | <.0001 | 0.0003 | 0.0002 | 0.0001 |
| SBM-C | SBM-C |  | 0.0016 | <.0001 | 0.0011 | 0.0008 | 0.0238 |
| ***Grower vs. Developer phase*** | |  |  |  |  |  |  |
| **Grower** | **Developer** |  |  |  |  |  |  |
| SBM-A | SBM-A |  | 0.983 | 1.000 | 0.956 | 0.990 | 1.000 |
| SBM-A | SBM-B |  | 1.000 | 0.999 | 1.000 | 1.000 | 0.753 |
| SBM-A | SBM-C |  | 0.958 | 1.000 | 0.907 | 0.903 | 1.000 |
| SBM-B | SBM-A |  | 0.968 | 1.000 | 0.722 | 0.795 | 0.999 |
| SBM-B | SBM-B |  | 1.000 | 1.000 | 1.000 | 1.000 | 0.989 |
| SBM-B | SBM-C |  | 0.932 | 0.929 | 0.611 | 0.520 | 0.986 |
| SBM-C | SBM-A |  | 0.999 | 1.000 | 0.994 | 1.000 | 1.000 |
| SBM-C | SBM-B |  | 1.000 | 0.981 | 1.000 | 0.976 | 0.609 |
| SBM-C | SBM-C |  | 0.995 | 1.000 | 0.979 | 0.993 | 1.000 |

^1^SBM-A, -B, and -C are diets containing soybean meals derived from Manitoba (MB)-grown soybean. Different superscripts within a column (variable) are significantly different at *P*<0.05. *n* = 6 for all treatments except in the layer phase, where *n* = 5 (per replicate cage).
